# White Cord Syndrome as a Rare Complication Post Cervical Spinal Decompression Surgery: A Case Report

**DOI:** 10.7759/cureus.70304

**Published:** 2024-09-27

**Authors:** Antoniette Marie C Guerrero, Vishal Prasad

**Affiliations:** 1 General Surgery, Ashford and St. Peter's Hospitals NHS Foundation Trust, Chertsey, GBR; 2 Trauma and Orthopaedics, Ashford and St. Peter's Hospitals NHS Foundation Trust, Chertsey, GBR

**Keywords:** myelomalacia, spinal decompression, ischemia reperfusion injury, white cord syndrome, spinal surgery complications

## Abstract

White cord syndrome (WCS) is a rare complication following spinal decompression surgery, characterized by acute neurological deterioration and T2 hyperintensity on MRI. This is a case of a 60-year-old female with cervical myelopathy and significant cord compression who developed WCS after anterior cervical decompression and fusion (ACDF) at C5/6 and C6/7. Preoperatively, she presented with gait disturbances, dexterity issues, and left-sided weakness, progressively worsening over two years. Postoperatively, she experienced significant deterioration, with new motor deficits on the right upper limb and urinary retention. Despite initial improvements, she developed bowel incontinence and a complete loss of power in her left leg three weeks post surgery. Magnetic resonance imaging (MRI) excluded recurrent cord compression but showed progressive myelomalacia. Diagnosed with WCS, she was treated with high-dose dexamethasone, resulting in gradual neurological improvement.

White cord syndrome should be a main differential diagnosis in patients with unexplained neurological deterioration following cervical spinal decompression surgery, especially those with chronic cord compression. Further research is needed to better understand the pathophysiology and risk factors of WCS to improve outcomes.

## Introduction

Cervical spondylotic myelopathy (CSM) is caused by chronic compression or inadequate blood supply to the cervical spinal cord due to cervical disc herniation, spinal stenosis, or instability. Clinically, it presents with neurological deficits that could lead to paralysis and death [1]. Symptoms typically involve gait disturbance, dexterity abnormalities, extremity weakness, paresthesia, and bladder/bowel abnormalities. This condition requires decompression surgery to prevent progression and reverse the neurologic deficits [2]. 

Typical causes of worsening neurologic function after surgical decompression include epidural hematoma, inadequate decompression, misplaced hardware, and/or contact with the cord or nerve roots. This phenomenon is a result of a reperfusion injury coined as “white cord syndrome” (WCS) [2], characterized by sudden motor weakness and the new signal change evident in a postoperative magnetic resonance imaging (MRI) [3,4,5]. While the underlying mechanism is not fully understood, it is theorized to occur because of the sudden blood flow or reperfusion, which causes damage to the spinal cord with ischemic damage after decompressive surgery like discectomy, laminectomy, or laminoplasty [2].

This paper presents a case of WCS where the patient experienced neurologic deterioration after an anterior cervical decompression and fusion (ACDF) of C5/6 and C6/7. With only a handful of WCS cases reported worldwide [1-6], this case report aimed to provide awareness on this very rare condition of white cord syndrome, its clinical presentation, diagnosis, and management to emphasize it as a possible complication after spinal cord decompression surgery. 

## Case presentation

**Figure 1:**
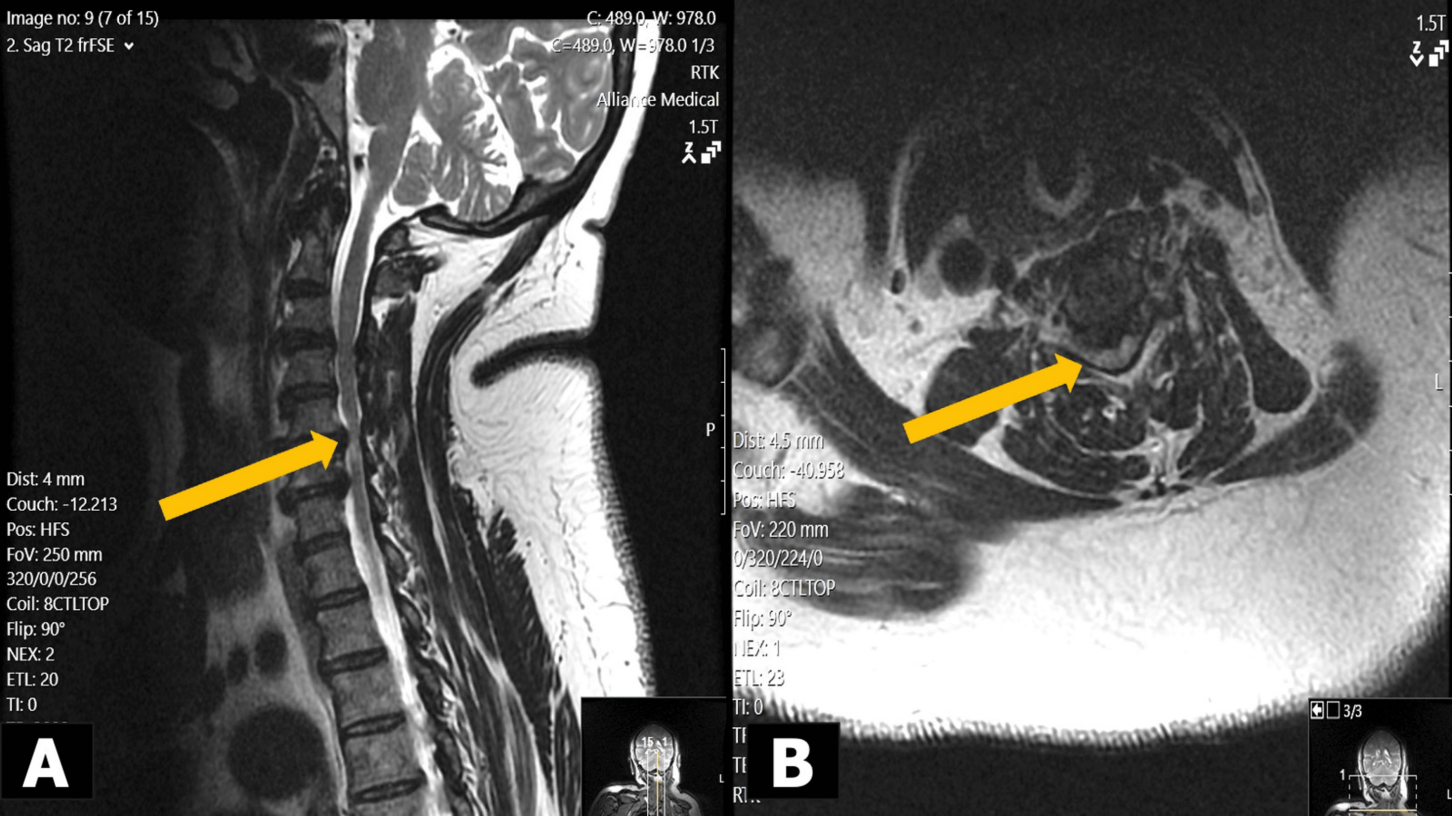
Preoperative T2-weighted MRI scans showing multilevel cervical foraminal narrowing with multilevel nerve root impingement of the right C4 and left C5 nerve roots (yellow arrows) in a) sagittal view and b) axial view.

A 60-year-old female presented in the clinic with reduced mobility, gait disturbance, left-sided weakness, and dexterity issues for two years. She was first diagnosed with cervical myelopathy in 2021 and went away to think about the operation for some time. She presented in the clinic in 2021 with an ataxic gait, a positive Romberg’s test, and power that was 5/5 on both upper and lower limbs but had globally brisk reflexes, inverted brachioradialis, and signs of cord compression. She noticed the worsening hand function with pain in both arms and reduced mobility for two years. She also had difficulty lifting her left foot more than her right foot. Her medical history included hypertension, well-controlled asthma, transient ischemic attack (TIA), and type 2 diabetes mellitus. On examination, she was in a wheelchair, and her gait, balance, and left-sided limb weakness worsened. This was initially thought to be a stroke; however, that was excluded by necessary investigations and scans. She was profoundly ataxic and showed positive Romberg’s, Hoffman’s, and Lhermitte’s signs. Her left side was more affected than the right side globally, with evident left-sided clawing and small muscle wasting. There was global hyporeflexia and three beats of clonus on the left side, and no clonus on the right. Preoperative MRI showed central disc bulges at C5/C6 and C6/C7 with myelomalacia and multilevel cervical foraminal narrowing with possible multilevel nerve root impingement, particularly on the right C4 and left C5 nerve roots. (Figures 1a, 1b). 

She then underwent a two-level ACDF of C5/6 and C6/7. The procedure was successful without any adverse events, with the intraoperative findings as expected from the preoperative imaging. 

**Figure 2:**
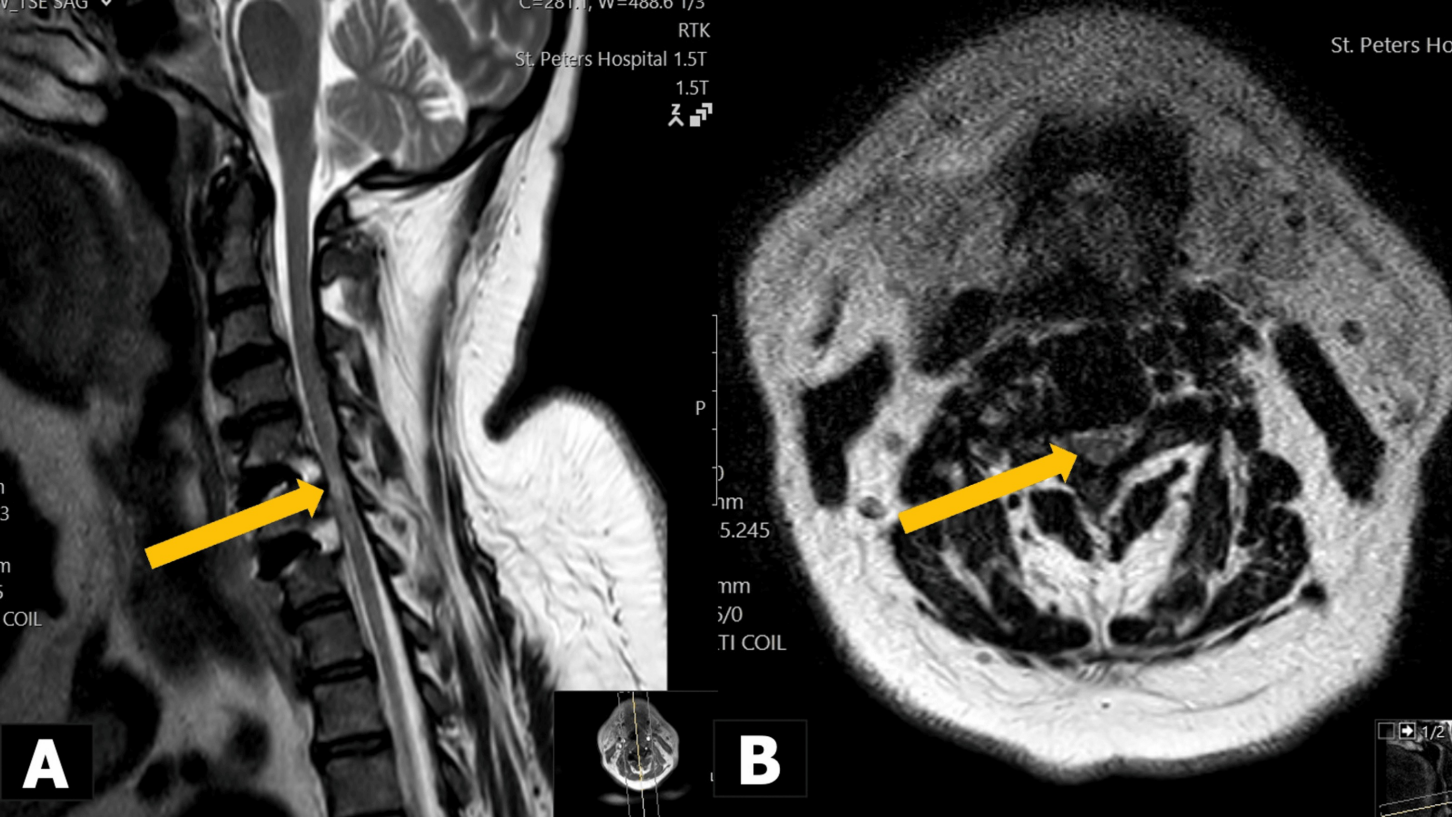
Initial postoperative T2-weighted MRI scans showing emphasis on the progressive myelomalacia at C5/C6 and C6/C7 (yellow arrows) but no recurrent cord compression in a) sagittal view and b) axial view.

Prior to surgery, her symptoms were predominantly on the left side. However, postoperatively, she significantly deteriorated and had a progressive loss of fine motor control on her right upper limb with mildly reduced power alongside a new symptom of urinary retention. She could not mobilize, was hoist-dependent, and was then catheterized as she developed postoperative urinary retention. This resolved a few days later, and the urinary catheter was removed. Eight days post-op, the patient developed bowel incontinence, and deterioration was noticed to be worse. A postoperative MRI was done, which showed no recurrent cord compression (Figures 2a, 2b). On the third week post-op, the patient had completely lost power in her left leg. 

**Figure 3:**
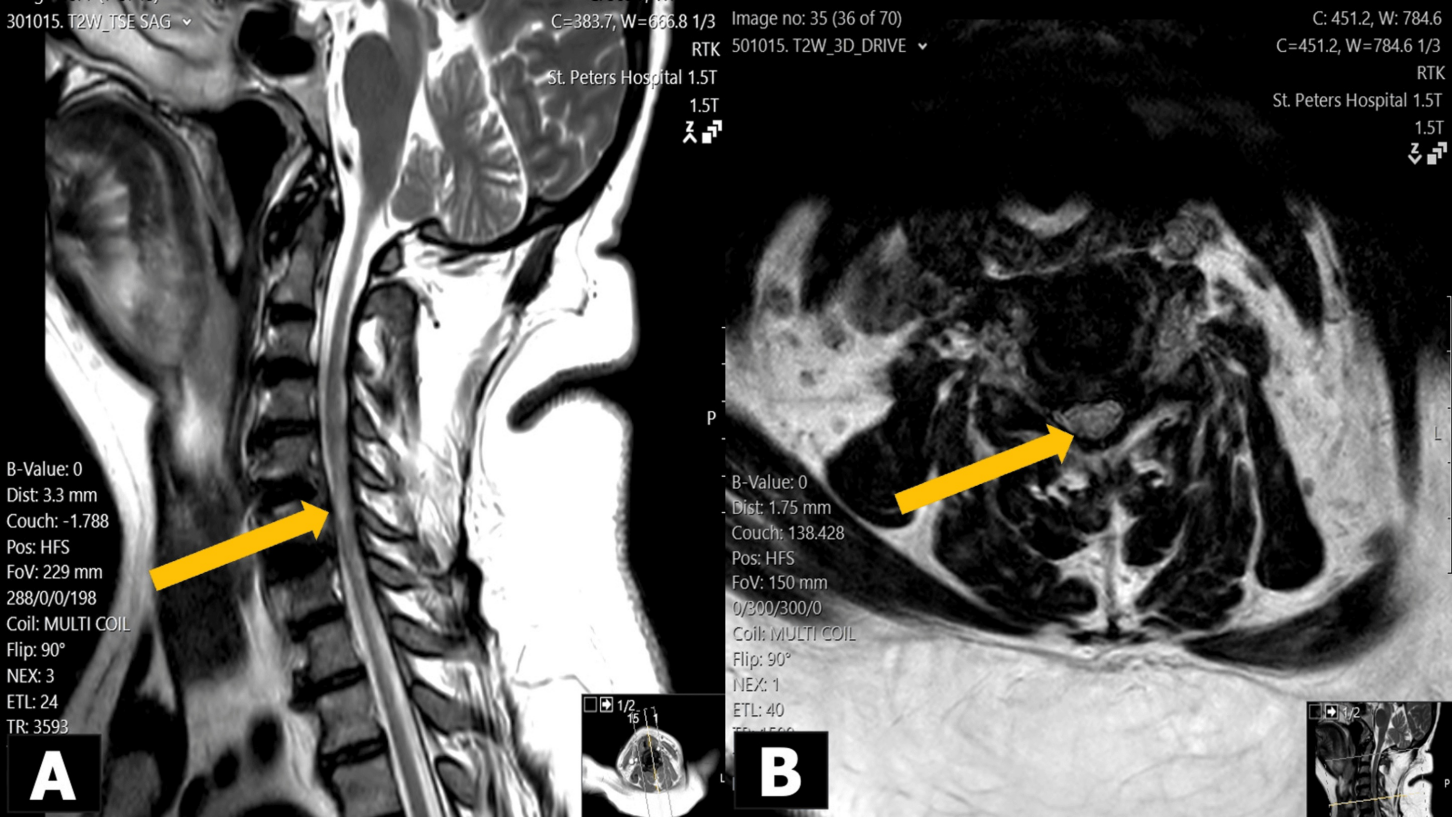
Postoperative T2-weighted MRI scans after six weeks showing persisting myelomalacia most prominent at C5/C6 level (yellow arrows) but with no acute cord compression in a) sagittal view and b) axial view.

Six weeks post-op, the MRI of the cervical spine was repeated, which still showed multilevel degenerative changes with progressive myelomalacia at C5/C6 and C6/C7 but no recurrent cord compression (Figures 3a, 3b). There were bilateral exiting foraminal stenoses at multiple levels. She continued to have physiotherapy, and the pain was managed accordingly.

After confirming that it was WCS, the patient was placed on IV dexamethasone therapy for about two weeks according to protocol [7]. She received 16 mg once daily or 8 mg twice daily for days one to three, then tapered slowly thereafter to 8 mg once daily on days four to six, 4 mg once daily on days seven to nine, and 2 mg once daily on days 10-12. Upper limb neurology slowly improved since receiving dexamethasone, and she was advised to mobilize as tolerated. She remained in the orthopedic ward while on dexamethasone therapy and awaited rehabilitation transfer. 

The patient developed new-onset homonymous left inferior quadrantanopia almost two months post ACDF. The MRI of the head with diffusion-weighted magnetic resonance imaging (DWI) and MR angiography (MRA) were requested. The MRI showed an acute right posterior cerebral artery (PCA) infarct, and the MRA showed no thrombus, a patent circle of Willis, a patent proximal right posterior cerebral artery, and a vertebrobasilar system. The stroke team reviewed her and advised an ultrasound Doppler of the carotid arteries, which showed no hemodynamically significant stenosis in either extracranial carotid tree. She was given 300 mg of aspirin daily for two weeks and was eventually switched to Clopidogrel 75 mg as her lifelong medication. 

The patient was sent to a neurorehabilitation unit almost three months after the surgery given her complications in the ward, but she managed her pain and continued her rehabilitation with much improvement in her balance and in her left arm movement/power. Her quadrantanopia gradually improved as well. Upon discharge, she was sent home with a long-term catheter and district nurse follow-up for catheter management. She will also be followed up in the neurorehabilitation unit within three months from her discharge to update with the new MRI and also to have a stroke team follow-up.

## Discussion

Acute paralysis following spinal surgery could be attributed to epidural hematoma, fixation failure, iatrogenic cord injury, incomplete decompression, and vascular compromise. The first case of ischemic reperfusion injury was reported initially by Chin et al. in 2013 after an anterior cervical spine decompression and termed “white cord syndrome” due to the characteristic T2 hyperintensity on a postoperative MRI scan [8-10].

White cord syndrome is usually diagnosed after a cervical spine decompression surgery, where it has been hypothesized to occur when blood flow is restored to previously ischemic tissues and organs that cause oxygen-free radical release leading to neuronal damage and disruption to the blood-spinal cord barrier, hence called ischemic-reperfusion injury [9]. 

It is a diagnosis of exclusion characterized by an increased intramedullary cord signal on a postoperative T2 weighted image (T2WI) MRI scan. Preoperative MRI can clarify the site and type of cervical disc herniation as well as the damage to the spinal cord and nerve roots, providing a reference point to guide surgeons on the treatment options and prognosis of cervical spondylosis. A postoperative MRI can exclude WCS if hematoma, spinal cord compression, or intraoperative spinal cord injury have been detected [2]. 

The patient had severe stenosis and central disc bulges at C5/C6 and C6/C7 with myelomalacia and developed sensory and motor deficits on postoperative day eight following C5/6 and C6/7 ACDF. On the third postoperative week, the patient had completely lost power in her left leg. In this case, it is evident that MRI scans have excluded cord compression, but the delayed presentation could most likely be due to chronic myelomalacia prior to surgery. The timing of decompression plays a significant role in the presentation of WCS as it has been associated with over-expression of cytokines, microglial activation, and astrogliosis, which can exacerbate reperfusion injury [9]. 

Previously published cases have presented neurological deficits either during surgery or in the early postoperative period. However, late-onset neurological deficits were also reported, with varying degrees of severity among cases [1, 9]. Risk factors such as advanced age, comorbidities, and chronic cord compression have been hypothesized to cause WCS, given that a higher degree of inflammatory response would be evident in this group of patients [2]. In our case, the late-onset WCS could be caused by the patient’s chronic hypertension and chronic cord compression, knowing that the former can cause atherosclerosis and endothelial damage secondary to decreased production and increased nitric oxide (NO) degradation. As NO is significant to maintain adequate blood flow, reduced levels could cause vasoconstriction, reducing perfusion to the spinal cord. Decreased NO can also contribute to endothelial dysfunction, contributing to inflammation and edema. After decompression surgery, the chronically ischemic tissue would now allow blood to flow from the area, causing dysfunction of autoregulatory mechanisms of arteries during the ischemic period, with rapid adenosine triphosphate (ATP) loss, cell membrane depolarization, and intracellular calcium accumulation. A sudden increase in blood flow after the reperfusion leads to an increase in oxidative stress, thereby overproducing reactive oxygen species (ROS) and eventually, cell death [4]. 

Our patient had a two-level ACDF of C5/6 and C6/7, which may have caused a top-down reflow similar to a case presented by Giammalva et al. (2017), where they inferred the chronically hypoxic cervical cord could have led to the worsening of the existing ischemia [4]. Moreover, it is advisable to maintain blood pressure intra- and postoperatively to maintain the spinal cord perfusion to reduce the risk of spinal cord injury/ischemia.

With the emerging rise of more than 10 WCS cases reported in the literature, all of these were managed with high-dose steroids, as these can reduce oxidative stress together with physical rehabilitation. In our patient's case, her symptoms have greatly improved after a high dose of dexamethasone over two weeks. 

## Conclusions

Common causes of neurological deficits following cervical spinal decompressive surgery are usually due to hematoma or iatrogenic injury. A high index of suspicion should be warranted in an unexplained neurological deterioration post-spinal decompression in a case that has had chronic cord compression prior to surgery. A postoperative MRI is key to its diagnosis, as it has shown a consistent finding among all the WCS case reports. Once identified, the treatment of choice is high-dose steroids alongside neurorehabilitation to aid in the patient’s recovery. Further research is needed to understand its pathophysiology and risk factors to guide surgeons to properly assess it and hopefully determine the appropriate management to address this rare complication, especially in patients presenting with chronic cord compression.
